# Supplementary material for: Partial cortico-hippocampectomy in cats, as therapy for refractory temporal epilepsy: A descriptive cadaveric study
Source: PLoS One. 2021 Jan 15;16(1):e0244892. doi: 10.1371/journal.pone.0244892 (PMC7810294; doi:10.1371/journal.pone.0244892)
Supplement: Attachment — Submitted filename: Rebuttal letter.pdf [file pone.0244892.s001.pdf]

Dear Mr Cozzi,

I would like to thank you for taking the time to reviewing our study. I very much appreciated your comments and ameliorations and I have already integrated them into the paper. I would also use this opportunity to respond to you and discuss each of the mentioned points.

Reviewer #1: The article of Zilli and Coll. describes a new and fairly accurate approach to partial cortico-temporal surgery for the treatment of refractory temporal epilepsy in cats.
The article is well written, well-documented and innovative. I have no objection to the rationale and core of the text.

Authors: I am extremely glad to hear that you like our approach for the resection of the hippocampus in cats with antiepileptic drug resistant temporal epilepsy.

Reviewer #1: The AA repeatedly discuss the potential “damage” of surrounding brain areas induced by the proposed surgery. In my opinion this way to express the situation is wrong, as they are proposing and discussing a cadaver study. There is no “damage”, but a potential trespassing of the intended area defined as a target. I would rather use a different wording of their description. This is no simple whim, as I had to read the abstract and text accurately to understand the meaning they give to the “damage”.
Matter of fact, the potential real damage induced by the procedure in living animals is probably greatly due to edema and inflammation of the surrounding tissue, and not by excessive removal of the target. Please comment

Authors:

We absolutely agree that the negative effects of tissue manipulation would be more profound in a living animal and may rather include intraparenchymal haemorrhage and edema.

We would like to describe the potential complications of brain tissue manipulation while removing the hippocampus. Therefore, we have tried to improve the wording in the text in order to support the reader´s understanding of our concept of “damage”. Moreover, we added a paragraph in the results including further complications we encountered during the study and that can occur also in living animals.

After thoroughly studying the wording, we cannot see a semantic difference between primary mechanical damage of the tissue and the secondary effects after mechanically destroying/manipulating the tissue, which can also be described as damage, as general term. The word injury has a synonymous meaning. Nevertheless, we made some specifications, hoping to improve the comprehension of the text.

Reviewer #1: Although they describe the external approach in some detail, in my opinion they may have underestimated the huge adverse effect of the surgery on the temporal muscle and related structure of the area. Considering the importance of the said muscle and temporo-mandibular joint in cats, I suggest to examine the potential negative consequence.

Authors:

Although we see the concern of the reviewer, the lateral transtemporal approach to the brain is a standard technique in veterinary neurosurgery and the mechanical insult to the temporal muscle in our approach seems to be relatively minor compared with the described basolateral approaches that include resection of the zygomatic arch, of the condylar and/or coronoid processes of the mandibula. We do not resect the muscle or widely detach its insertion or impair the vascular supply. The temporo-mandibular joint remains unaffected. However, we have included a paragraph in the discussion evaluating this concern.

Reviewer #1: The AA described the experimental series of cats, that includes very small (young) individuals. A descriptive and detailed Table would help

Authors:

We have added the requested table in the section "Material and methods".

Reviewer #1: The AA should also cite the classic stereotaxic atlas of the cat brain by Snider et al. and later partial books on the same subject. Stereotactical surgery is way less invasive. I understand that the therapeutic approach envisioned here requires extensive tissue removal. However, the pros and cons of selective ablation or coagulation of limited brain areas focal to epilepsy induction should be at least briefly discussed.

Authors:

We added the requested references, as well as a paragraph in the discussion reflecting on the use of neuronavigational systems and its advantages and disadvantages. We are aware that a less invasive approach would be possible if a histological examination is not required, nevertheless we consider it as an extremely important point in patients with structural epilepsy. Indeed, also in human medicine the examination of the resected specimen is always performed.

We hope you will appreciate the changes we apported to the paper and we look forward to your reply.

Kind regards,

Jessica Zilli
